# miR-200a Targets *PITX2* to Mediate Goose Fibroblast Proliferation Through the Wnt Pathway

**DOI:** 10.3390/ani15213171

**Published:** 2025-10-31

**Authors:** Shuyu Jiao, Hongyuan Yang, Heng Ge, Shaomei Li, Suozhou Yang, Chunyan Mou

**Affiliations:** 1 College of Animal Science and Technology, Southwest University, Chongqing 402460, China; jiaosy0105@163.com (S.J.); 18385933151@163.com (H.Y.); geheng114514@163.com (H.G.); suozhouyang@126.com (S.Y.); 2 National Center of Technology Innovation for Pigs, Chongqing 402460, China; shaomeili123@163.com; 3 Chongqing Key Laboratory of Herbivore Science, Chongqing 402460, China

**Keywords:** miRNA, PITX2, Wnt/β-catenin pathway, goose down, skin development

## Abstract

The economic value of goose down is attributed to its extensive application in the production of down-based clothing and related products. The primordium formation stage governs the proper morphogenesis of the feather follicle, while the Wnt signaling pathway serves a positive regulatory function during this stage. To identify critical miRNAs and molecular mechanisms regulating the development of goose feather follicle primordium, we performed transcriptomic sequencing of skin tissues collected from six geese at pre- and post-feather follicle primordium developmental stages. Bioinformatics analysis identified 350 differentially expressed miRNAs (DE miRNAs), which were functionally enriched in processes related to system development and multicellular organismal development, etc. As demonstrated by dual-luciferase reporter experiments, miR-200a binds directly to *PITX2*’s 3′ untranslated region (3′UTR). Furthermore, overexpression of miR-200a decreased the expression levels of genes linked to the Wnt pathway and suppressed the proliferation of GEDFs, as validated by RT-qPCR, CCK8, and EdU assays. Notably, co-transfection experiments demonstrated that miR-200a-mediated regulation of GEDF proliferation through the Wnt pathway is functionally dependent on *PITX2*. Collectively, this work expands the regulatory network underlying feather follicle development and provides a genetic foundation aimed at breeding geese with enhanced down production quality.

## Simple Summary

Goose down possesses significant economic value due to its exceptional softness and warmth properties. miRNAs have been demonstrated to indirectly influence feather follicle morphology, growth, and development through the targeted regulation of genes or pathways associated with hair/feather follicle formation. However, the current research on miRNAs in hair follicle (HF) development mainly focuses on the proliferation and differentiation of HF stem cells and the process of HF regeneration, and there are fewer studies on miRNAs in the process of early development of feather follicles, so it is worthy of in-depth research. Our findings reveal that miR-200a relies on *PITX2* to mediate the Wnt pathway in order to regulate the proliferation of goose embryonic dermal fibroblasts (GEDFs) and thus may regulate goose feather follicle development. Collectively, this work expands the regulatory network underlying feather follicle development and provides a genetic foundation aimed at breeding geese with enhanced down production quality.

## 1. Introduction

Goose down possesses significant economic value due to its exceptional softness and warmth properties, making it an ideal material for down garments, quilts, and related products. The feather follicle, from which down arises, proceeds through three stages: induction, organogenesis, and cytodifferentiation. The morphogenetic process initiates when epidermal cells undergo localized thickening to form placodes following their migration within the skin regions. Epithelial-derived signaling molecules subsequently induce dermal mesenchymal cells to aggregate, forming the dermal condensate (DC). This epithelial–mesenchymal interaction establishes the primordium, which, through subsequent proliferation and differentiation, gives rise to feather buds. These buds ultimately develop into mature feather follicles and associated feathers or down [1].

Multiple signaling pathways are involved in the morphogenetic development of HFs. Substantial evidence demonstrates that the Wnt pathway functions throughout the developmental stages. Study of murine models underscores the pathway’s importance, as *β-catenin* knockout disrupts primordium formation and abrogates normal morphogenesis during the inductive phase of HF [2]. Wnt/β-catenin signaling controls the rapid transition from progenitor to DC cells [3]. At the stage of HF primordium development, Wnt pathway ligands activate dermal papillary cell (DPC) proliferation and thus promote HF development. For example, *Wnt10b* promotes HF growth and DPC proliferation in Rex rabbits through canonical Wnt signaling activation [4,5]. Similarly, through the Wnt/β-catenin pathway, *CRABP2* and *SOX18* both promote DPC proliferation in sheep [6,7]. At the post-transcriptional level, miRNA-181a-5p has been shown to promote HF morphogenesis through its regulatory effects on Wnt/β-catenin signaling in DPC [8], and miRNA-214 regulates skin and HF development by Wnt pathway activity [9].

The ectodysplasin receptor (EDAR), a TNF receptor superfamily member, mediates EDA signaling during skin development. The EDA/EDAR/NF-κB pathway exhibits functional crosstalk with Wnt/β-catenin signaling during the initiation and maintenance of HF primordium [10]. Alterations in EDAR signaling influence the onset of various HF types, hair shaft development, and sebaceous gland morphology [11]. The bone morphogenetic protein (BMP) ligands inhibit HF fate by suppressing *Edar* expression in the epidermis, and treatment with Noggin, an antagonist of BMP, leads to a higher density of HF placodes [12]. Research indicates that *Bmp2*, *Bmp4*, and *Bmp7* inhibit the development of hair and feather follicle placodes [13,14,15,16]. The fibroblast growth factor (FGF) signaling pathway is essential for HF morphogenesis, primarily through its interaction with FGFR. *Fgf20*, expressed specifically in HF primordium, serves as a positive developmental regulator, as evidenced by the absence of DC formation in *Fgf20* mutant mice [17]. Beyond molecular signaling, cellular movements are essential for proper morphogenesis. Real-time automated imaging and 3D reconstruction revealed that during placode formation, inner cells migrate anteriorly while outer cells undergo posterior displacement, transforming an initially disk-like radial pattern to an asymmetric bud-like structure [18].

Some genes are key regulators in HF development, which may be regulated through signaling pathways. *PITX2*, a member of the RIEG/PITX homeobox gene family, encodes a crucial transcription factor involved in multiple developmental processes. Initially characterized as the causative gene for Rieger syndrome [19]. *PITX2* has been recognized to play broader roles in human disease, hypothalamic-pituitary axis development, and organ symmetry establishment. Using *Pitx2* knockout mice, it was demonstrated that deletion of *Pitx2* delays epidermal invagination, reduces progenitor cell proliferation and dental epithelial cell differentiation, and that the interaction of *Pitx2*, *Sox2*, and *Lef1* modulates the signaling of dental epithelial cell transduction centers and dental epithelial stem cell homeostasis [20,21]. PITX2 activates Wnt signaling, while *Dact2* diminishes this activation and affects tooth epithelial development [22]. *Pitx2* induces the expression of miR-200a-3p, which in turn inhibits the expression of *Pitx2* and *β-catenin*, promoting mesenchymal-to-epithelial differentiation [23]. The *Pitx2*-miR-200c/141-*Noggin* regulation pathway is crucial for dental epithelial differentiation [24]. Pitx2 expression was detected in human epidermis and HF, and Pitx2 induced the expression of keratins 16 and 17 in ORS cells, supporting its positive regulatory role in HF differentiation [25]. Notably, in Inner Mongolian Cashmere Goat, chi-miR-21-5p negatively regulates *PITX2*, potentially influencing secondary HF development [26].

The molecular mechanisms driving the early development of goose feather follicles are not well understood. Based on the economic value of goose down, the development of the feather follicle in geese needs to be studied urgently. In order to explore the miRNAs and molecular mechanisms that regulate the early stage of goose feather follicle, skin tissues from the stage before and after the formation of the primordium were selected for sequencing, and the DE miRNAs were screened by data analysis. miR-200a and *PITX2* were used for the functional validation and regulatory network studies in GEDFs. In summary, our study elucidated the regulatory role of miR-200a/*PITX2* in the proliferation of GEDFs and provided the molecular regulatory mechanism of miR-200a/*PITX2*/Wnt in the early development of goose feather follicles.

## 2. Materials and Methods

### 2.1. Utilization of Experimental Animals and Procedures for Sample Collection

All 150 eggs from Sichuan white geese were collected from a healthy, vaccinated, and disease-free breeding flock from the poultry farm at Chongqing Academy of Animal Science. The geese were selected because they are a dominant indigenous breed in Sichuan Province, China, known for their high down production. This sample size is also consistent with those commonly used in published poultry science studies of a similar nature. Eggs were incubated under standard conditions (38 °C ± 0.2 °C, 70% ± 2% relative humidity) until reaching target developmental stages, which were determined based on the timing of feather follicle development established through morphological examination in our previous study. Embryonic skin tissues were collected during two critical morphogenetic stages, including pre-primordium formation and post-primordium, with three biological replicates per time point. Three biological replicates are standard, ensuring sufficient statistical power and accounting for natural population variation. Part of the collected samples were preserved at −80 °C until RNA was extracted for the transcriptome sequencing process.

### 2.2. Primary GEDF Cell Isolation and Culture

Goose embryos at developmental stages E9-E11 were dissected and promptly moved to phosphate-buffered saline (**PBS**), which is ice-cold. Using sterile microdissection techniques, we removed the cephalic, extremities, and visceral organs, retaining only the embryonic torso for subsequent processing. The remains were minced into 1 mm^3^ tissue fragments using sterile surgical scissors. Following one PBS wash and centrifugation, tissue fragments were enzymatically digested in 0.25% trypsin solution for 30 min at 37 °C with mild stirring. The digestion was terminated by adding culture medium, and the cell suspension was dissociated through repeated pipetting. After filtration and centrifugation, DMEM/F-12 media containing 12% fetal bovine serum (**FBS**) and 1% penicillin-streptomycin were used to resuspend the cells. The primary cells were seeded into culture flasks and maintained under standard conditions (37 °C, 5% CO_2_) with medium changes every 24 h. Cell viability was assessed using the Trypan Blue exclusion method immediately after isolation. The average viability of the isolated cells was consistently found to be over 90%, confirming that the isolation procedure yielded viable cells. We detected the expression levels of *Vimentin* and *Col1a1* via RT-qPCR to validate that the isolated cells were GEDFs.

### 2.3. DE miRNAs Analysis and Target Genes Prediction

DESeq2 was chosen for differential expression analysis due to its statistical rigor, robustness with small samples, and built-in multiple testing correction. miRNAs meeting the threshold criteria of adjusted *p* < 0.05 and |log_2_ fc| ≥ 1 were statistically defined as DE miRNAs. The adjusted *p*-value was calculated using the Benjamini–Hochberg procedure to control the false discovery rate (FDR). For target gene prediction of identified DE miRNAs, we employed the miRanda v3.3a software. We applied the following criteria: a targeting score > 140 and a maximum free energy < −10. The 3′UTR sequences required for target prediction were extracted using TBtools v1.089 software. Reference cDNA sequences were obtained from the Ensemble database (http://ftp.ensembl.org/pub/release-115/fasta/anser_brachyrhynchus/cdna/Anser_brachyrhynchus.ASM259213v1.cdna.all.fa.gz (10 January 2022).

### 2.4. GO Annotation and KEGG Pathway Enrichment

Comprehensive functional annotation of DE miRNA target genes was performed using the gene ontology database, encompassing molecular function (**MF**), cellular component (**CC**), and biological process (**BP**). First, we quantified the total number of target genes mapped to a specific functional term. Statistical significance of enrichment was subsequently determined through hypergeometric testing, using false discovery rate (**FDR**) adjustment for multiple comparisons (*p* < 0.05). Pathway analysis was conducted using the KOBAS web server, which similarly employed hypergeometric testing to identify significantly enriched KEGG pathways (*p* < 0.05). This approach enabled systematic identification of both molecular functions and potential regulatory pathways involved in feather follicle development.

### 2.5. RNA Oligonucleotides and Cell Transfection

To examine the function of miR-200a in goose feather follicle morphogenesis, we designed and synthesized the following oligonucleotides through GenePharma (Shanghai, China): (1) miR-200a mimics, (2) mimics negative control (mimics NC), (3) miR-200a inhibitor, and (4) inhibitor negative control (Inhibitor NC). Complete nucleotide sequences are provided in Table 1. Transfection experiments were initiated when primary GEDFs reached 70–80% confluence. Following the manufacturer’s instructions, all transfections were conducted utilizing the Lipofectamine 2000 transfection reagent (Thermo Fisher Scientific, Waltham, MA, USA). Transfection efficiency was confirmed for each experiment. For miRNA mimics/inhibitors, it was quantified 24 h post-transfection by measuring miRNA expression relative to negative controls, with no significant cytotoxicity observed at the concentrations used.

### 2.6. Dual Luciferase Reporter Gene Assay

In order to functionally validate the targeting interaction of miR-200a and *PITX2*, we engineered two luciferase reporter constructs: a wild-type (***PITX2*-WT**) vector containing the native miR-200a binding sequence within the *PITX2* 3′UTR, and a mutant (***PITX2*-MT**) vector with site-directed mutations in the predicted binding site. Both constructs were synthesized and cloned into the pmirGLO vector (Sangon, Shanghai, China). For the assay, 293T cells were co-transfected in 24-well plates with four groups: (1) mimics NC + *PITX2*-WT, (2) miR-200a mimics + *PITX2*-WT, (3) mimics NC + *PITX2*-MT, and (4) miR-200a mimics + *PITX2*-MT, with four biological replicates per group. Luciferase activity was evaluated 48 h after transfection using the Dual Luciferase Reporter Gene Assay Kit (Beyotime Biotechnology, Shanghai, China). Firefly luciferase activity was normalized to Renilla luciferase (pRL-TK) to control for transfection and cell number variations, and expressed as a Firefly/Renilla ratio.

### 2.7. Extraction of Total RNA with Real-Time Quantitative Polymerase Chain Reaction (RT-qPCR)

For miRNA expression profiling, the RNAiso Plus reagent (Takara Bio Inc., Shiga, Japan) was used to extract total RNA from the skin tissues of goose embryos. The One Step miRNA cDNA Synthesis Kit (HaiGene, Harbin, China) was applied for subsequent cDNA synthesis, and TB Green Premix Ex Taq II FAST qPCR master mix (Takara Bio) was used for RT-qPCR. The small nuclear RNA U6 served as the endogenous control for normalization. Parallel mRNA expression analysis was conducted using RNA extracted with RNAiso Plus (Takara Bio), and RevertAid Reverse Transcription Premix (Thermo Fisher Scientific, Waltham, MA, USA) was used to reverse transcribe it. Total RNA quality and quantity were rigorously assessed: purity was confirmed by NanoDrop (A260/A280 = 1.9–2.1), and integrity by 1.5% agarose gel electrophoresis. RT-qPCR was performed under identical conditions as the miRNA analysis, with GAPDH serving as the reference gene. qRT-PCR was performed with three biological replicates, each run in technical triplicate. Table 1 lists all of the primer sequences used in these analyses. The amplification efficiency for each primer pair was validated prior to formal experiments.

### 2.8. Cell Counting Kit-8 (CCK8) Assay

The CCK-8 was used to measure cell proliferation quantitatively (Meilun Biotechnology Co., Ltd., Dalian, China). GEDFs were seeded in 96-well plates, which could be transfected with respective plasmids at 60% confluence, with eight biological replicates and eight technical replicates per group. Proliferation assays included a blank (medium only), negative control (NC, cells with control miRNA), and treated groups. Prior to the experiment, the CCK8 reagent was mixed and incubated for two hours at 37 °C. Absorbance measurements at 450 nm were performed at 12, 24, 36, and 48 h post-transfection using a microplate reader. Absorbance was blank-corrected, normalized, and expressed as percent viability relative to the negative control (set to 100%).

### 2.9. 5-Ethynyl-2′-deoxyuridine (EdU) Assay

EdU incorporation was used to quantitatively measure cell proliferation. GEDFs were seeded, which could be transfected with respective plasmids at 60% confluence, with six biological replicates per group. Twenty-four hours post-transfection, cells were exposed to 50 μM EdU (RiboBio, Guangzhou, China) for 2 h at 37 °C. Following fixation and permeabilization, EdU detection was performed in accordance with the protocol. All samples were imaged using the same exposure settings by an inverted fluorescence microscope. Six randomly selected fields of view (20× objective) were captured per replicate to ensure representative sampling. The percentage of EdU-positive cells was quantified using ImageJ v1.51 software, applying uniform thresholding parameters across all groups. The proliferation rate is presented as the percentage of EdU-positive cells relative to the total number of Hoechst-stained nuclei in the same field of view.

### 2.10. Data Analysis

All experimental data were evaluated utilizing Student’s *t*-test by SPSS v22.0 software to assess group differences after confirming normality (Shapiro–Wilk) and homogeneity of variance (Levene’s test). For multiple comparisons, Bonferroni correction was applied, with a corrected *p* < 0.05 considered significant.

## 3. Results

### 3.1. Differential Expression of miRNAs Before and After the Stage of Primordium Formation

**Figure 1:**
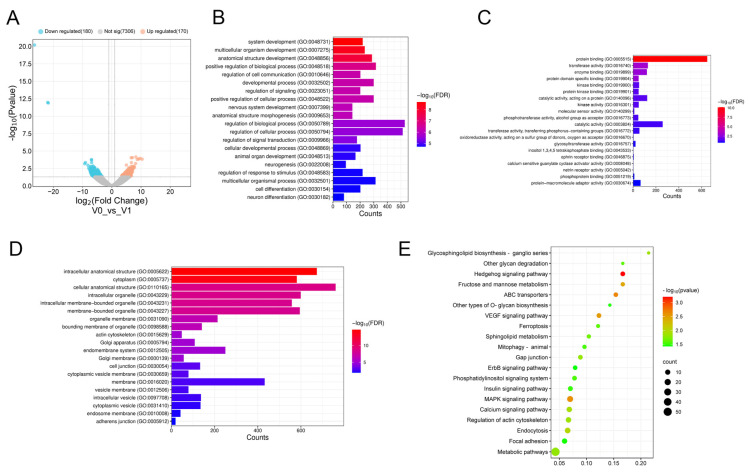
Analysis and function annotation of DE miRNAs during goose feather follicle primordium development. (**A**) Volcano plot illustrating the distribution of DE miRNAs between pre-primordium (V0) and post-primordium (V1) stages. Red points indicate significantly upregulated miRNAs, blue points represent significantly downregulated miRNAs, and gray points denote miRNAs without significant expression changes. (**B**–**D**) The top 20 highly enriched terms in biological processes, molecular functions, and cellular components are displayed by GO enrichment analysis of miRNA target genes. The y-axis lists the individual GO terms, and the x-axis shows the number of miRNAs enriched in each term. (**E**) KEGG pathway enrichment analysis showing the top 20 significantly enriched pathways. The color gradient represents the enrichment significance from red (most significant) to green (least significant). The color gradient indicates enrichment significance, ranging from red (most significant) to green (least significant), while the size of the dots reflects the quantity of miRNAs associated with each pathway. miRNAs mapped to each pathway. Color intensity represents normalized expression levels, where red denotes high expression and blue signifies low expression in relation to the mean.

The formation of primordium represents a critical developmental stage in feather follicle morphogenesis, with stagnation at this stage resulting in developmental abnormality. To investigate the molecular mechanisms underlying this process, we performed transcriptomic analysis of skin tissues collected during pre- and post-primordium formation stages. Sequencing results revealed 350 significantly DE miRNAs (|log_2_FC| > 1, *p* < 0.05), comprising 170 upregulated and 180 downregulated miRNAs (Figure 1A).

### 3.2. GO Annotation and KEGG Pathway Analysis of DE miRNAs

To characterize the biological process and signaling pathways associated with DE miRNAs, comprehensive GO and KEGG pathway enrichment analyses were conducted on the predicted target genes. The GO analysis revealed significant enrichment in several biological processes, including multicellular organism development, system development, and regulation of cell communication, and so on (Figure 1B). Molecular function analysis showed predominant enrichment for protein binding, transferase activity, protein domain-specific binding, and so on (Figure 1C). Cellular component analysis identified significant enrichment in intracellular anatomical structures, cytoplasm, adherens junctions, and so on (Figure 1D). KEGG pathway analysis implicated several critical pathways in feather follicle development, including the Hedgehog, MAPK, and calcium signaling pathways, and so on (Figure 1E). In addition, we observed specific enrichment patterns in developmental processes: 11 genes were associated with skin development and morphogenesis, 6 genes with hair follicle development and morphogenesis, and 16 genes with epidermal development and cell differentiation. Furthermore, pathway analysis revealed substantial involvement of key developmental signaling pathways: 13 genes exhibited enrichment within the Wnt signaling pathway, 10 genes in the FGF signaling pathway, and 2 genes in the SHH signaling pathway. These pathways collectively contribute to feather follicle development and morphogenesis.

### 3.3. Expression Detection of DE miRNAs in Embryonic Skin Tissues of Geese

**Figure 2:**
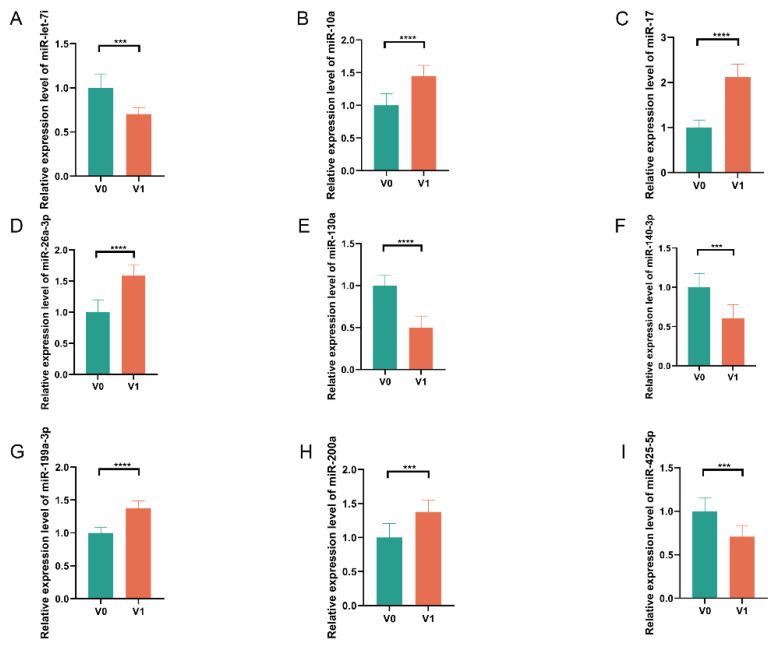
Validation of miRNA sequencing results by RT-qPCR analysis. (**A**–**I**) RT-qPCR validation of nine selected DE miRNAs confirmed the reliability of our sequencing data. All measurements were performed with three biological replicates, with data presented as mean ± SEM (*** *p* < 0.001, **** *p* < 0.0001).

Nine potential miRNAs (miR-let-7i, miR-10a, miR-17, miR-26a-3p, miR-130a, miR-140-3p, miR-199a-3p, miR-200a, and miR-425-5p) were chosen for quantitative verification using RT-qPCR in order to validate the miRNA sequencing results. These miRNAs were analyzed in skin tissues of geese at both pre- and post-primordium formation stages. The RT-qPCR results demonstrated expression patterns consistent with our sequencing data (Figure 2A–I), thereby confirming the reliability of the miRNA-seq findings.

### 3.4. miR-200a Negatively Regulates PITX2

**Figure 3:**
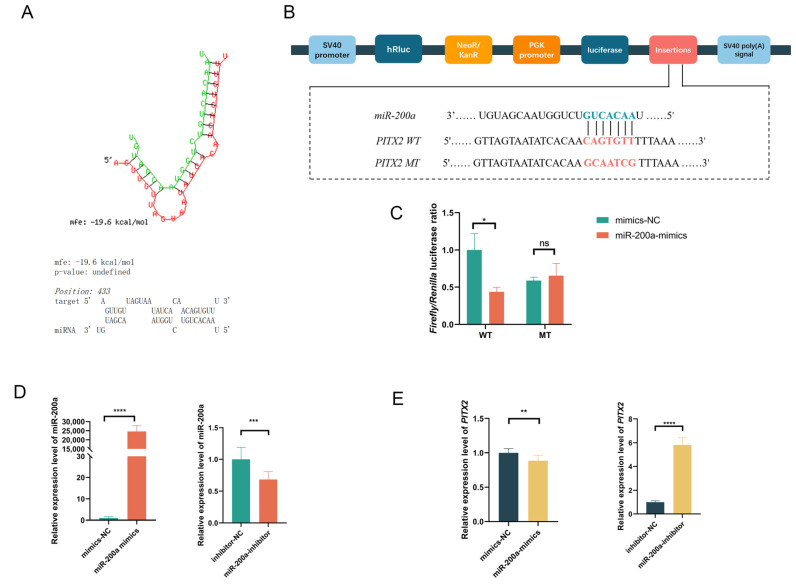
miR-200a regulates *PITX2* expression by targeting the 3′UTR of *PITX2*. (**A**) Computational prediction of miR-200a binding regions within the *PITX2* 3′UTR using RNAhybrid, showing minimum free energy hybridization. (**B**) Schematic representation of PITX2-WT and PITX2-MT constructs used for validation, with mutated nucleotides highlighted in red. (**C**) Dual-luciferase reporter gene assay demonstrating miR-200a-mediated suppression of *PITX2* 3′UTR activity. (**D**) Expression of miR-200a was detected by RT-qPCR after its overexpression and silencing. (**E**) Corresponding *PITX2* mRNA expression were detected after miR-200a overexpression and silencing by RT-qPCR (n = 3, * *p* < 0.05, ** *p* < 0.01, *** *p* < 0.001, **** *p* < 0.0001, ns = not significant). All data represent mean ± SEM from three independent experiments.

Based on our miRNA sequencing analysis, we selected *PITX2* from the predicted target genes of miR-200a for further study (Figure 3A). To experimentally validate this regulatory relationship, we performed dual-luciferase reporter gene assays in 293T cells. Cells undergo co-transfection with wild-type (**WT**) and mutant (**MT**) *PITX2* 3′UTR reporter constructs along with miR-200a mimics or mimics negative control (**mimics NC**), followed by fluorescence activity measurement at 48 h post-transfection (Figure 3B). The results demonstrated distinct patterns between the MT and WT groups. The relative fluorescence activity of the miR-200a and mimics NC did not differ significantly in the MT group (*p* > 0.05). In contrast, the WT group showed significantly reduced relative fluorescence activity following miR-200a mimics treatment compared to mimics NC (*p* < 0.05) (Figure 3C). These findings provide direct evidence that miR-200a specifically binds to the 3′UTR of *PITX2*, and this interaction leads to post-transcriptional repression of *PITX2* expression.

### 3.5. Overexpression of miR-200a Reduces the Expression of Genes Related to the Wnt Pathway and Inhibits the Proliferation of GEDFs

**Figure 4:**
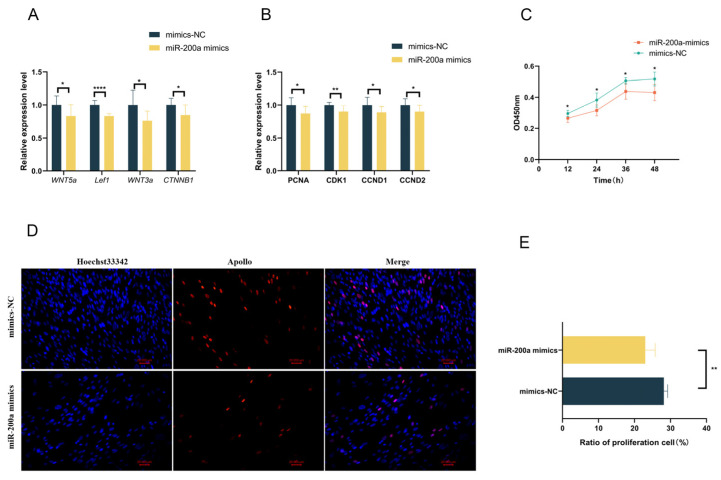
Overexpression of miR-200a reduces the Wnt signaling and inhibits the proliferation of GEDFs. (**A**) RT-qPCR analysis demonstrating significant downregulation of Wnt signaling pathway genes following miR-200a overexpression. (**B**) RT-qPCR detection of proliferation-related gene expression after overexpression of miR-200a. (**C**) CCK8 detection of cell proliferation of GEDFs after overexpression of miR-200a at 12 h, 24 h, 36 h, and 48 h. (**D**) Images of EdU assays at 24 h post-transfection, with EdU-positive cells (red) counterstained with DAPI (blue). (**E**) Quantification of EdU-positive cells demonstrates significantly reduced proliferation rates in the miR-200a mimics group. All experiments were performed with three biological replicates (n = 3), with data presented as mean ± SEM (* *p* < 0.05, ** *p* < 0.01, **** *p* < 0.0001).

The Wnt signaling pathway positively influences feather follicle development. To further investigate whether miR-200a regulates the expression of genes associated with the Wnt pathway and the proliferation of GEDFs, we conducted gain-of-function experiments by transfecting GEDFs with miR-200a mimics or NC mimics. RNA extraction and RT-qPCR at 24 h post-transfection revealed successful miR-200a overexpression (Figure 3D), accompanied by significant downregulation of *PITX2* expression (Figure 3E). The results indicated that the overexpression of miR-200a substantially reduced expression levels of key Wnt pathway genes (*WNT5a*, *LEF1*, *WNT3a,* and *CTNNB1*; Figure 4A) and cell proliferation marker genes (*PCNA*, *CDK1*, *CCND1,* and *CCND2*; Figure 4B). Then, CCK-8 assays showed significantly lower OD450 values in miR-200a-treated groups compared to controls (Figure 4C). In addition, the EdU cell proliferation assay was employed to further demonstrate the impact of miR-200a on the proliferation of GEDFs. Similarly, the quantity of EdU-positive cells was markedly reduced in the miR-200a mimics-treated group in comparison to the mimics NC group (Figure 4D,E). These consistent results from multiple experimental approaches demonstrate that miR-200a overexpression suppresses both Wnt pathway activity and GEDF proliferation.

### 3.6. Silencing miR-200a Expression Elevated the Expression of Genes Associated with the Wnt Signaling Pathway and Promoted the Proliferation of GEDFs

**Figure 5:**
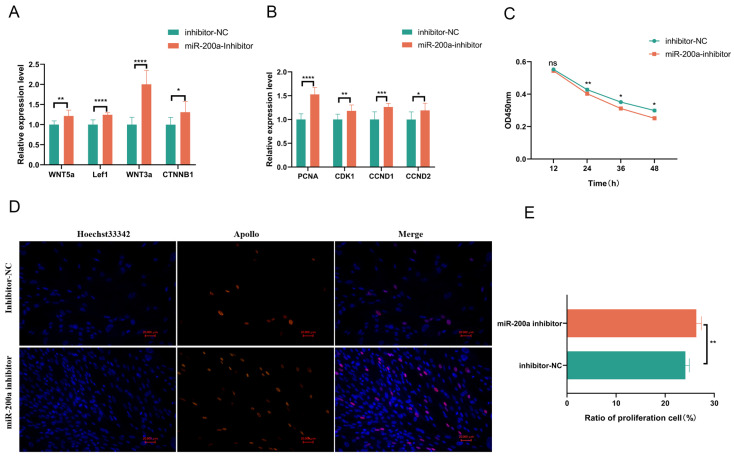
Silencing of miR-200a enhances Wnt signaling and promotes GEDF proliferation. (**A**) RT-qPCR analysis showing significant upregulation of Wnt pathway genes following miR-200a inhibition. (**B**) Increased expression of proliferation markers in miR-200a-inhibited cells. (**C**) The CCK-8 assay indicates increased cell proliferation in groups treated with the miR-200a inhibitor relative to control groups. (**D**) EdU assay was performed at 24 h post-transfection for cell proliferation. (**E**) Quantitative analysis revealed significantly higher EdU-positive cell ratios in miR-200a-inhibited groups. All experiments included three biological replicates (n = 3), with data presented as mean ± SEM. (* *p* < 0.05, ** *p* < 0.01, *** *p* < 0.001, **** *p* < 0.0001, ns = not significant).

We conducted loss-of-function experiments by transfecting GEDFs with an inhibitor of miR-200a or an inhibitor negative control (**Inhibitor NC**) in order to investigate the functional effects of miR-200a silencing. RNA extraction and RT-qPCR at 24 h post-transfection confirmed successful miR-200a silencing (Figure 3D), which was accompanied by significant upregulation of *PITX2* (Figure 3E). Experiments revealed that blocking miR-200a substantially enhanced the expression of genes involved in the Wnt pathway and key cell proliferation regulators (Figure 5A,B). Furthermore, the results of the CCK8 experiment demonstrated that the group treated with miR-200a inhibitors had a considerably higher OD450nm value than the Inhibitor NC group (Figure 5C). Additionally, the EdU assay results demonstrated that, in contrast to the Inhibitor NC group, the EdU-positive cells in the miR-200a Inhibitor-treated group had a significantly higher rate (Figure 5D,E). Therefore, silencing miR-200a could increase the expression of genes associated with the Wnt pathway and promote the proliferation of GEDFs.

### 3.7. Overexpression of PITX2 Increases the Expression of Genes Associated with the Wnt Signaling Pathway and Promotes the Proliferation of GEDFs

**Figure 6:**
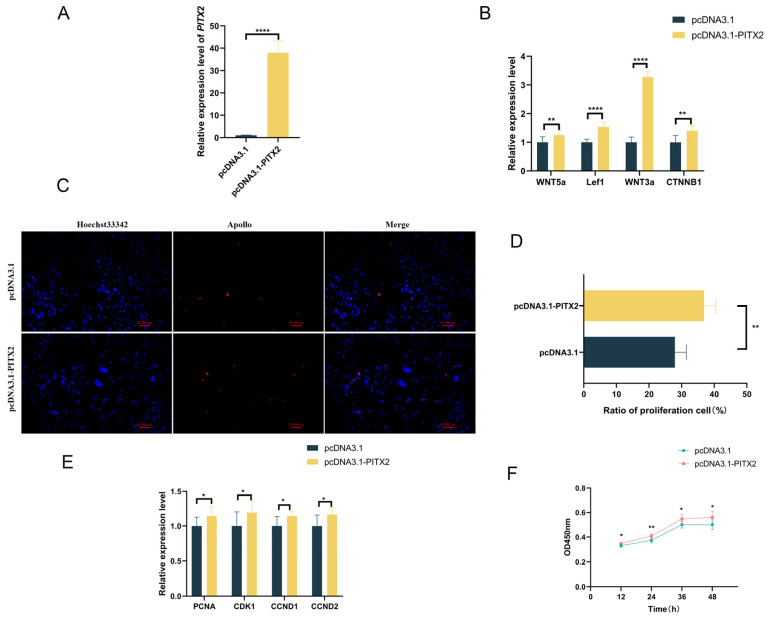
*PITX2* overexpression activates Wnt signaling and enhances GEDF proliferation. (**A**) RT-qPCR detection of *PITX2* expression after its overexpression. (**B**) RT-qPCR detection of Wnt pathway gene expression after overexpression of *PITX2*. (**C**) An EdU assay was performed for cell proliferation (**D**). Quantitative analysis demonstrating increased EdU-positive cell ratios in *PITX2*-overexpressing groups. (**E**) RT-qPCR for the expression of proliferation-related genes after overexpression of *PITX2*. (**F**) CCK8 detection of cell proliferation of GEDF after overexpression of *PITX2*. All experiments were conducted with three biological replicates (n = 3), with data presented as mean ± SEM. (* *p* < 0.05, ** *p* < 0.01, **** *p* < 0.0001).

To elucidate the function of *PITX2*, the target gene of miR-200a, in regulating Wnt signaling and GEDF proliferation, we constructed a *PITX2* overexpression plasmid and transfected the pcDNA3.1-*PITX2* plasmid into GEDFs. RT-qPCR analysis confirmed successful *PITX2* overexpression (Figure 6A), which led to significant upregulation of Wnt pathway-related genes (Figure 6B). Concomitantly, we observed increased expression of cell proliferation marker genes (Figure 6E). CCK-8 analysis demonstrated significantly higher OD450 values in *PITX2*-overexpressing cells compared to the pcDNA3.1 group (Figure 6F), while EdU assays revealed a greater proportion of EdU-positive cells following *PITX2* overexpression (Figure 6C,D). These results collectively demonstrate that *PITX2* enhances both Wnt pathway activity and proliferative capacity in GEDFs.

### 3.8. Incorporation of pcDNA3.1-PITX2 Rescued the Phenotypes Caused by Overexpression of miR-200a

**Figure 7:**
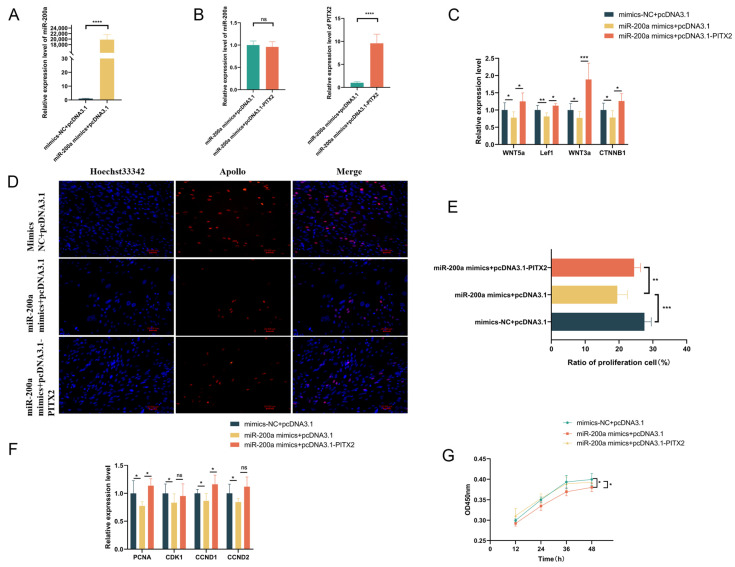
Overexpression of *PITX2* rescues the effects of miR-200a. (**A**) The expression levels of miR-200a were assessed with RT-qPCR after co-transfection of miR-200a mimics with pcDNA3.1. (**B**) Expression levels of miR-200a and *PITX2* were assessed with RT-qPCR after co-transfection of miR-200a mimics with pcDNA3.1-*PITX2*. (**C**) The expression of Wnt pathway genes was detected following co-transfection using RT-qPCR. (**D**,**E**) EdU was performed 24 h after co-transfection to detect cell proliferation and count the positive cell rate. (**F**) The expression of proliferation-related genes was detected following co-transfection using RT-qPCR. (**G**) CCK8 detection of cell proliferation in GEDFs after co-transfection. All experiments included three biological replicates (n = 3), with data presented as mean ± SEM. (ns *p* > 0.05, * *p* < 0.05, ** *p* < 0.01, *** *p* < 0.001, **** *p* < 0.0001).

Whether the regulation of Wnt signaling pathway-related gene expression and GEDF proliferation by miR-200a is dependent on *PITX2*. To explore this problem, we performed rescue experiments through co-transfection of pcDNA3.1-*PITX2* and miR-200a mimics in GEDFs. Successful overexpression was confirmed for all experimental groups: mimics NC + pcDNA3.1 (control), miR-200a mimics + pcDNA3.1 (miR-200a OE), and miR-200a mimics + pcDNA3.1-*PITX2* (rescue group) (Figure 7A,B). Experiment analysis revealed that *PITX2* overexpression in the rescue group significantly restored expression levels of Wnt pathway-related genes compared to the miR-200a OE group (Figure 7C). Similarly, cell proliferation marker genes show different levels of recovery (Figure 7F). CCK-8 and EdU assays consistently demonstrated that incorporation of pcDNA3.1-*PITX2* reversed the proliferative inhibition caused by miR-200a overexpression (Figure 7D,E,G). In conclusion, the addition of pcDNA3.1-*PITX2* rescued the phenotypes of Wnt signaling pathway-related genes and GEDF proliferation caused by overexpression of miR-200a.

## 4. Discussion

Current research on HF has primarily focused on humans, mice, chickens, and cashmere goats. Despite the widespread utilization of high-quality goose down feathers, the molecular mechanisms governing feather follicle development remain poorly characterized. Down feather production is regulated by the cyclic development of feather follicles, progressing through growth, regression, and resting phases [27]. During the embryonic period, the development of the down undergoes three stages: induction, organogenesis, and cytodifferentiation, and the process is influenced by many factors, such as genes, non-coding RNAs, and signaling pathways [1]. In this study, transcriptome sequencing of skin tissues before and after primordium formation revealed 350 significantly DE miRNAs, indicating that these miRNAs might be crucial for the stage of primordium development. Functional annotation revealed enrichment in key pathways, including Hedgehog, MAPK, and calcium signaling, with predominant involvement in multicellular organism development, system development, and cellular communication regulation. Particularly, the Wnt/β-catenin signaling pathway is critical in the early development of the feather follicle. Our study identified the pathway genes (including *WNT1* and *WNT6*) as targets of DE miRNAs during feather follicle primordium formation. *WNT1*, which exhibits specific expression in the dermis and primordium, has been previously demonstrated to stimulate differentiation of dermal progenitor cells and promote cellular proliferation [28]. Several genes, including *SOSTDC1*, *EGFR*, and *PITX2*, exhibited high enrichment in biological processes related to skin and HF development. *SOSTDC1* plays a crucial role in appendage morphogenesis, with studies demonstrating that its overexpression affects limb development and reduces the size of placodes in mammary glands [29]. *EGFR* is essential for mediating epithelial–mesenchymal interactions during HF development. Similarly, *PITX2* contributes to the developmental regulation of various skin appendages, including teeth and mammary glands. Additionally, some signaling pathways involved in HF development, such as FGF and SHH, were enriched among DE miRNAs target genes. These findings suggest that *SOSTDC1*, *EGFR*, *PITX2,* and enriched pathways may have conserved functions in the early stages of goose feather follicle development, similar to their functions in mammalian HF formation.

miRNAs typically modulate gene expression at the post-transcriptional stage, thus affecting animal growth and development. Emerging evidence has identified numerous miRNAs participating in hair/feather follicle morphogenesis. For instance, miR-140-y/*TCF4* has been implicated in feather follicle regulation in Hungarian white geese, while miR-144-y modulates feather follicle development in Zhedong white geese through targeting *FOXO3* [30,31]. However, current studies on miRNAs in HF development mainly focus on the proliferation and differentiation of HF stem cells as well as the process of hair follicle regeneration, leaving little research regarding miRNA functions during early inductive stages of feather follicle formation, so it is worthy of more attention. In this study, miRNA–mRNA interactions were examined to screen for factors involved in early feather follicle development in geese. Among the differentially expressed candidates, miR-200a emerged as a particularly interesting target due to its evolutionary conservation across species and previous studies in HF biology. Supporting evidence includes its identification in androgenetic alopecia (AGA) microarray studies and computational predictions for sheep HF development [32,33]. Transcriptomic profiling in our work revealed significant differential expression of miR-200a during primordium formation, a finding validated by subsequent RT-qPCR analysis. Functional characterization demonstrated that miR-200a overexpression downregulates key Wnt pathway genes (*WNT5a*, *LEF1*, *WNT3a*, and *CTNNB1*) and suppresses GEDF proliferation. These collective results establish miR-200a as a negative regulator of primordium formation, potentially acting through modulation of Wnt signaling cascades. This discovery provides novel insights into the molecular mechanisms governing early feather follicle development.

Pan-genomic analyses have identified variable genes enriched in the “follicle maturation” GO term, suggesting their potential contribution to interspecies variation in feather characteristics among geese [34]. *PITX2* is involved in the early development of skin appendages such as teeth. Shared genes and pathways during the early stage of development often yield similar phenotypes across different skin appendages [35]. The findings indicated that *PITX2* overexpression led to a marked enhancement in the expression of Wnt pathway-associated genes and a substantial rise in the proliferation of GEDFs. This implies that *PITX2* may have a positive regulatory function in the early stages of the feather follicle development. To verify whether miR-200a mediates the Wnt pathway through *PITX2* to regulate the proliferation of GEDFs, we conducted rescue experiments through co-transfection of miR-200a mimics and pcDNA3.1-*PITX2*. Quantitative analyses revealed that *PITX2* supplementation effectively reversed the miR-200a-mediated suppression of both Wnt signaling activity and GEDF proliferation. These results were consistently validated through CCK-8 and EdU assays.

A limitation of this study is the sample size used for the transcriptomic analysis. Although the three biological replicates per group meet the minimum requirement for differential expression analysis and initial discovery, a larger cohort would enhance the statistical power and generalizability of the findings. Future studies will focus on validating these results in larger populations. The DE miRNAs and their target genes identified in this study hold potential for development into molecular markers for marker-assisted selection. In future breeding programs, these markers can be utilized to screen individuals with high down potential during early developmental stages of geese, thereby accelerating genetic progress.

**Figure 8:**
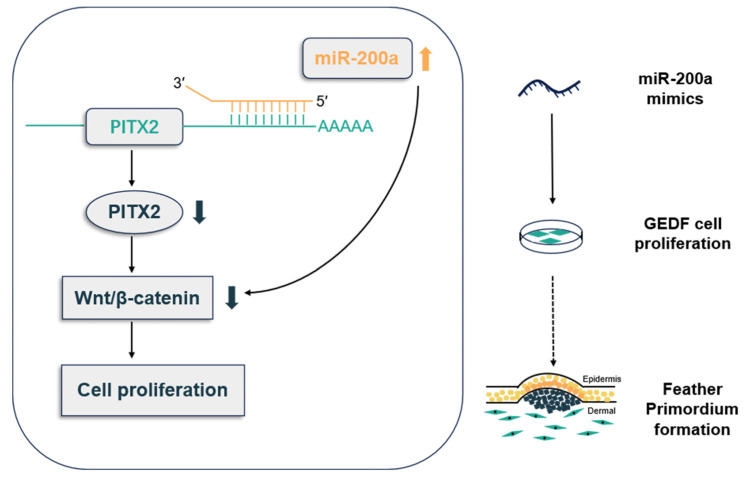
Molecular mechanism of miR-200a-mediated regulation during early goose feather follicle development in GEDFs.

Our findings suggest that miR-200a directly binds to *PITX2*, leading to downregulation of Wnt pathway genes and consequent inhibition of GEDF proliferation. Furthermore, we propose a mechanistic model whereby miR-200a-mediated suppression of cellular proliferation may impair the formation of the primordium, thereby regulating feather follicle development in geese (Figure 8). These results offer a new understanding of the molecular mechanisms governing goose feather development and establish miR-200a as a critical regulator of feather follicle morphogenesis through its modulation of the *PITX2*-Wnt signaling axis. Despite employing recognized prediction algorithms and stringent screening criteria, computational predictions inherently carry a false positive rate. Therefore, the predicted DE miRNAs in this study require validation through subsequent experiments to confirm their reliability. We note that although our in vitro cell model provides valuable mechanistic insights, it cannot fully recapitulate the complexity of the in vivo environment, which may impact the application of our results.

## 5. Conclusions

Through comprehensive transcriptomic analysis, our study identified DE miRNAs potentially involved in regulating goose feather follicle primordium development. We experimentally validated that miR-200a directly targets binding to *PITX2*, establishing a functional regulatory relationship. *PITX2* overexpression activates Wnt pathway gene expression and enhances GEDF proliferation. These findings collectively reveal that miR-200a relies on *PITX2* to mediate the Wnt pathway in order to regulate the proliferation of GEDFs and thus may regulate goose feather follicle primordium genesis (Figure 8). This work advances our understanding of the molecular networks governing early goose feather follicle development and provides valuable genetic insights for breeding aimed at improving both the quantity and quality of goose down. Future studies may integrate gene editing technologies to further investigate candidate genes involved in regulating feather follicle development.

## Figures and Tables

**Table 1:** Oligonucleotide sequences and RT-qPCR primers in this study.

Name	Sequence (5′-3′)
mimics NC	UUGUACUACACAAAAGUACUG
miR-200a mimics	UAACACUGUCUGGUAACGAUGU
Inhibitor NC	CAGUACUUUUGUGUAGUACAA
miR-200a inhibitor	ACAUCGUUACCAGACAGUGUUA
miR-26a-3p	F: CCTATTCTTGGTTACTTGCACTG
	R: CAGGTCCAGTTTTTTTTTTTTTT
let-7i	F: TGAGGTAGTAGTTTGTGCTGTT
	R: CAGGTCCAGTTTTTTTTTTTTTT
miR-199a-3p	F: ACAGTAGTCTGCACATTGGTT
	R: CAGGTCCAGTTTTTTTTTTTTTT
miR-140-3p	F: TACCACAGGGTAGAACCACGG
	R: CAGGTCCAGTTTTTTTTTTTTTT
miR-130a	F: CAGTGCAATGTTAAAAGGGCAT
	R: CAGGTCCAGTTTTTTTTTTTTTT
miR-10a	F: TACCCTGTAGATCCGAATTTGTG
	R: CAGGTCCAGTTTTTTTTTTTTTT
miR-17	F: ACTGCAGTGAAGGCACTTGTAGCAT
	R: CAGGTCCAGTTTTTTTTTTTTTT
miR-425-5p	F: AATGACACGATCACTCCCGTTGAG
	R: CAGGTCCAGTTTTTTTTTTTTTT
miR-200a	F: TAACACTGTCTGGTAACGATGT
	R: CAGGTCCAGTTTTTTTTTTTTTT
U6	F: TACAGAGAAGATTAGCATGG
	R: CAGGTCCAGTTTTTTTTTTTTTT
PITX2-qPCR	F: TGCACACCATCTCCGACACCT
	R: CGCCGCTGCCTCTTCTTCTT
WNT5a-qPCR	F: CCAGCTCTTGGTGGTCTTTAG
	R: TCCTTGGGAAAGCCCTGCTA
LEF-1-qPCR	F: AGCCTTCTCATGCGGTTCACC
	R: AGGAGCTGGAGGATGTCTGGAC
WNT3a-qPCR	F: TGAACAGGCACAACAACGAAGC
	R: ACCACCAGCAGGTCTTCACTTC
CTNNB1-qPCR	F: CGTGAAGGCTTGTTGGCAATCT
	R: TGGCATAGAACAGCACGGAGTC
PCNA-qPCR	F: CAGCCATATTGGTGATGCAG
	R: GGTCAGTTGGACTGGCTCAT
CDK1-qPCR	F: GAAGTCGTGACGCTGTGGTA
	R: TTGTTGGGTGTCCCTAAAGC
Cyclin D1-qPCR	F: AGACCATCCGACGAGCCTAC
	R: TTCTGCTCCTCGCAAACCTCC
Cyclin D2-qPCR	F: ACCCCAAGAGCTGCTGGAATG
	R: TGGCACAAAGGGCGATGAAC
GAPDH-qPCR	F: AAGGGCATCCTGGCATACAC
	R: CATCAAGTCCACCACACGGT

## Data Availability

The datasets used and/or analyzed during the current study are available from the corresponding author on reasonable request.
